# Texting and Walking: Strategies for Postural Control and Implications for Safety

**DOI:** 10.1371/journal.pone.0084312

**Published:** 2014-01-22

**Authors:** Siobhan M. Schabrun, Wolbert van den Hoorn, Alison Moorcroft, Cameron Greenland, Paul W. Hodges

**Affiliations:** The University of Queensland, School of Health and Rehabilitation Science and National Health and Medical Research Council (NHMRC) Centre of Clinical Research Excellence in Spinal Pain, Injury and Health, Brisbane, Queensland, Australia; University of South Australia, Australia

## Abstract

There are concerns about the safety of texting while walking. Although evidence of negative effects of mobile phone use on gait is scarce, cognitive distraction, altered mechanical demands, and the reduced visual field associated with texting are likely to have an impact. In 26 healthy individuals we examined the effect of mobile phone use on gait. Individuals walked at a comfortable pace in a straight line over a distance of ∼8.5 m while; 1) walking without the use of a phone, 2) reading text on a mobile phone, or 3) typing text on a mobile phone. Gait performance was evaluated using a three-dimensional movement analysis system. In comparison with normal waking, when participants read or wrote text messages they walked with: greater absolute lateral foot position from one stride to the next; slower speed; greater rotation range of motion (ROM) of the head with respect to global space; the head held in a flexed position; more in-phase motion of the thorax and head in all planes, less motion between thorax and head (neck ROM); and more tightly organized coordination in lateral flexion and rotation directions. While writing text, participants walked slower, deviated more from a straight line and used less neck ROM than reading text. Although the arms and head moved with the thorax to reduce relative motion of the phone and facilitate reading and texting, movement of the head in global space increased and this could negatively impact the balance system. Texting, and to a lesser extent reading, modify gait performance. Texting or reading on a mobile phone may pose an additional risk to safety for pedestrians navigating obstacles or crossing the road.

## Introduction

Mobile phones are considered an essential part of everyday life, saturating all age groups and demographics. It is estimated that 77% of the world's population own a mobile phone and texting in particular, has emerged as a quick and cost effective method of communication. Although the dangers of typing text while driving have received considerable interest (e.g. [1], [2]), attention has only recently shifted to safety risks associated with texting while walking. For instance, individuals who type text while crossing the street in a virtual pedestrian environment experience more hits by motor vehicles, and look away from the street environment more frequently, than those who are not distracted [3]. Similarly, use of the email function on a mobile phone, which employs similar cognitive and manual demands as texting, reduces gait velocity, stride length and stance phase during walking [4]. These findings, coupled with a sharp increase in the number of pedestrians injured while talking or texting on a mobile phone since 2006 [5], have led to bans on texting while walking in some towns in the United States [6]. Yet despite the apparent danger of texting while walking, only one study has examined how texting affects gait kinematics.

Typing and reading text on a mobile phone may modify walking as a result of the increased cognitive demand placed on working memory and executive control [7] during performance of dual tasks, decreased availability of visual information of surroundings, or modified physical/mechanical demands associated with manipulation of the phone (e.g. requirement to maintain a stable relationship between eyes and phone in the hands), yet there is little data available to compare these challenges. Further, altered physical and cognitive demands as a result of the diverse uses of mobile phones (e.g. reading vs. typing text) may produce differing effects on gait performance. Lamberg and Muratori [8] recently demonstrated reduced walking speed and deviation from a straight path while typing text on a mobile phone, which they argued to be caused by cognitive distraction of a dual task. However, that study occluded vision of the floor and target with a hood that obscured all but the mobile phone from view and gait may have been altered by reduced availability of visual information (e.g. peripheral vision) rather than increased cognitive demands of texting. Typing text in natural circumstances preserves peripheral vision and this may be sufficient to guide an individual along a straight path at reasonable velocity, although effects may differ between typing and reading text.

To further explore the effects of mobile phone use on gait, we examined and compared the impact on gait performance and kinematics of typing and reading (without any manual input) text on a mobile phone when compared with walking without a mobile phone. We hypothesised that greater potential for cognitive distraction and modified mechanical demands associated with typing text would impact on gait performance to a greater degree than reading text.

## Methods

### Ethics statement

All procedures were approved by The University of Queensland Medical Research Ethics Committee and conformed to the declaration of Helsinki. All participants provided written, informed consent.

### Participants

Twenty-six healthy individuals (7 male; age 29±11 years; height 1.7±0.1 m; weight 71±13 kg, mean ± standard deviation) provided informed written consent to participate. Participants were excluded if they were less than 18 years of age, did not use a mobile phone with a touch screen and full QWERTY virtual keyboard, had less than 3 months experience with their current phone, did not use their phone on a daily basis, or if they had any neurological and/or musculoskeletal disorders that would interfere with gait. Participants were asked if they had experienced any previous accident while texting on their mobile phone and reported details regarding their typical mobile phone usage (Table 1).

**Table 1:** Demographic data and mobile phone usage.

Variable	Data
Handedness right ∶ left ∶ ambidextrous	24∶1∶1
Typing method one handed ∶ two handed ∶ either method	9∶15∶2
Phone orientation portrait ∶ landscape	22∶4
Phone type iphone ∶ other	21∶5
Usual use of autocorrect on ∶ off	22∶4
Months of current phone use (mean ± SD)	13.6±7.0
Number of minutes spent talking on a mobile phone per day (mean ± SD)	17.7±15.9
Number of minutes spent texting on a mobile phone per day (mean ± SD)	30.7±44.6
Number of subjects who reported prior texting related accidents	9

SD – standard deviation

### Procedure

Three experimental conditions were included: 1) walking at a comfortable pace, 2) walking at a comfortable pace while reading a passage on a mobile phone screen with minimal manual input other than scrolling through text [9], and 3) walking at a comfortable pace while typing the passage ‘the quick brown fox jumps over the lazy dog’. To standardise familiarity with the passage, participants texted the passage three times prior to data collection. Participants completed three trials of each condition and the order was randomised across participants.

In each condition participants walked in a straight line for ∼8.5 m. In the texting condition participants used their own mobile phone and their normal method of texting (one or two hands, phone held in portrait or landscape). No instruction was given regarding text accuracy and participants were free to correct their errors (or not) as they chose. However, autocorrect was turned off to allow the number of typing errors to be quantified. The number of errors was calculated as a proportion of the total words texted in each trial. The average number of correct words texted/minute was calculated.

### Gait kinematics

For movement registration, 8 cameras (T040, Vicon Motion Systems Ltd. Oxford, UK) were positioned at both sides of the walking path at ∼45 degree angle facing the direction of walking and placed ∼2 m apart. Clusters of three non-collinear reflective markers were attached to the back of the head using a head band and with double sided tape to the participant's body, at thorax (T6) and pelvis (posterior superior iliac spine). Single reflective markers were attached at the left and right heel. A reference measure, with the participant in the anatomical position facing the walking direction, allowed for alignment of cluster marker coordinate systems with the global coordinate system. The global coordinate system was defined with the positive *X*-axis in the walking direction, the positive *Y*-axis to the left, and the positive *Z*-axis upwards. Position data were filtered with a low pass 4^th^ order bi-directional Butterworth filter at 5 Hz. The sampling rate was set at 100 Hz.

### Data analysis

#### Basic gait parameters

Right heel strikes were determined from the local vertical minima of the heel marker [10]. *Stride time* was the time between consecutive heel strikes on the same side. *Stride length* was the distance between consecutive heel strikes on the same side. *Walking speed* was determined as the mean velocity of the pelvis in the walking direction.

#### Segment rotations

**Figure 1:**
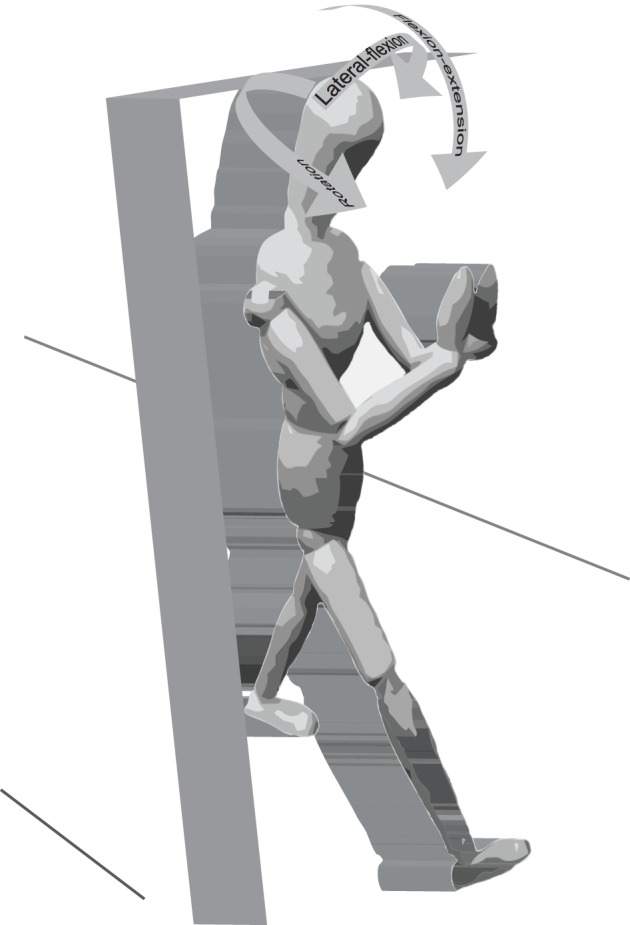
Definition of segmental movements.

Segment angles are reported as anatomically related movements (rotation (eq. 1), flexion-extension (eq. 2) and lateral flexion (eq. 3), Fig. 1), and were calculated from the segment axis system (x, y, z) in relation to the global axis system (x, y, z). The length of each segment axis was normalised to one.
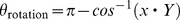
(1)

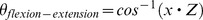
(2)

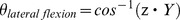
(3)


Relative motion between the thorax and head (neck motion), and between pelvis and thorax (trunk motion) were obtained by subtracting the time series of the relevant angles of the lower segment from the higher segment. Time series of segment angles were divided into stride cycles (from right heel strike to the following right heel strike). Within each stride cycle, the range of motion (ROM) was determined as the difference between the maximum and minimum angle, and was averaged across the stride cycles.

The average flexion angle of the head was determined as the mean of the flexion-extension time series.

#### Relative phase

Relative phase angle is a frequency domain measure, and provides information (in degrees) regarding the coordination between two segments' main component of motion (in this study; rotations at the same frequency as stride frequency) averaged over time. If two segments rotate in opposite directions, the relative phase angle is 180°, i.e. the coordination between two segments is ‘out-of-phase’. If two segments rotate together, the relative phase angle is 0°, i.e. the coordination is ‘in-phase’. The standard deviation of the relative phase angle is a measure of the spread of the relative phase angle around the mean phase. Relative phase between two segments (head – thorax and pelvis – thorax) of rotation, flexion-extension and lateral flexion movements was calculated as follows [11]: a windowed Fourier phase angle of the cross spectrum between the two segment angles was determined. This window was shifted 1 sample at a time, to allow an estimate of the continuous relative phase. The window length was set at 2.5 times the stride frequency. The average and standard deviation of the relative phase were determined with circular statistics.

#### Deviation from the straight-line

The average position in space of the pelvis cluster markers was used to determine deviations from a straight walking path. The straight line was defined by the position of two reflective markers placed at the beginning and end of the walking path, parallel to the walls of the room. All marker positions were rotated about the 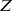
- axis by 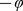 between the two markers that defined the straight line (
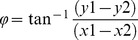
) to correct for any misalignment between the straight line and the 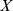-axis of the global axis system. The position of the pelvis at the time participants entered the volume (at which the markers were visible) were subtracted from the pelvis position. Deviations from the straight line were determined as: 1) Absolute distance from the straight line at the end of the walking path; and 2) total absolute distance travelled in medial-lateral direction divided by the total distance walked.

### Additional experiment – The impact of walking speed on gait kinematics with mobile phone use

To measure the motion of the phone/arm and to verify whether changes in kinematics were related to phone use and/or could be explained by the expected reduction in walking speed with phone use, 5 participants volunteered for an extra measurement on a separate day. To control for walking speed, this experiment was performed on a treadmill (Pioneer Pro, BH Fitness Products, California, USA) at two different speeds. The speeds were matched to that selected by the participant during the control and texting conditions of the main experiment. Kinematic data were collected as per the first experiment at both speeds while participants performed 3 tasks: control walking, reading and texting (randomised). To verify whether texting requires an additional mechanical demand above that required for reading (e.g. maintenance of phone position with respect to the head, fixation of the arms with the thorax), data of phone and elbow position were also collected using additional markers.

To test control of the position of the phone with respect to the head, the total distance moved by the phone (path) in three-dimensions per second was calculated: 1) in the global reference frame and 2) with respect to the head (after the coordinates of the phone were transformed into the head reference frame). To test whether arm movement was more constrained with respect to trunk motion (to hold phone still), the relative phase between the forward-backward arm movement and thorax rotations was calculated.

### Statistical analyses

All outcome variables were averaged across the three repetitions within each condition (walk; text; read). To ensure normal distribution, data were log transformed if Shapiro-Wilk test for normality was significant (*P*<0.05). All variables were compared between conditions with a repeated measures analysis of variance (ANOVA). The Greenhouse-Geisser correction was applied for suspected violation of independence of the repeated measures. Post hoc testing was conducted with Bonferroni correction. Statistics were performed in Stata (StataCorp LP, Texas, USA). Alpha level was set at *P*<0.05.

## Results

Demographic and mobile phone usage data are presented in Table 1. Nine of 26 (35%) participants reported a previous accident while texting on their mobile phone, including falls, trips and collisions with obstacles or other individuals. In the texting condition participants typed on average 7.9±2.8 words with an error rate of 3.5±3.1 words over the ∼8.5 m walked. Texting speed (number of words typed correctly per minute) was 23.0±9.4 words/minute.

### Basic gait parameters

Participants walked at a slower speed during reading and texting than when walking without the mobile phone, and walked slower during texting than reading (Table 2). Stride length and stride frequency were less during reading and texting than the control condition and less during texting than reading (see Table 2 for output of statistical analyses).

**Table 2:** Basic gait parameters.

	ANOVA_RM_			Post hoc analyses		Mean (±SD)		
Outcome measure	F ratio	P-value	Walk vs. Read	Walk vs. Text	Read vs. Text	Walk	Read	Text
Walking speed (m/s)	85.12	0.0000	0.0000	0.0000	0.0000	1.33 (0.15)	1.16 (0.14)	1.01 (0.17)
Stride length (m)	110.94	0.0000	0.0000	0.0000	0.0000	1.35 (0.12)	1.23 (0.09)	1.15 (0.09)
Stride frequency (Hz)	49.14	0.0000	0.0002	0.0000	0.0000	0.99 (0.06)	0.95 (0.08)	0.88 (0.11)
Abs path lateral direction (m)	13.23	0.0000	0.874	0.0000	0.0011	0.07 (0.02)	0.08 (0.02)	0.10 (0.03)
Delta right foot position (m/stride)	14.12	0.0000	0.0041	0.0000	0.2111	0.03 (0.01)	0.04 (0.01)	0.04 (0.01)

**Figure 2:**
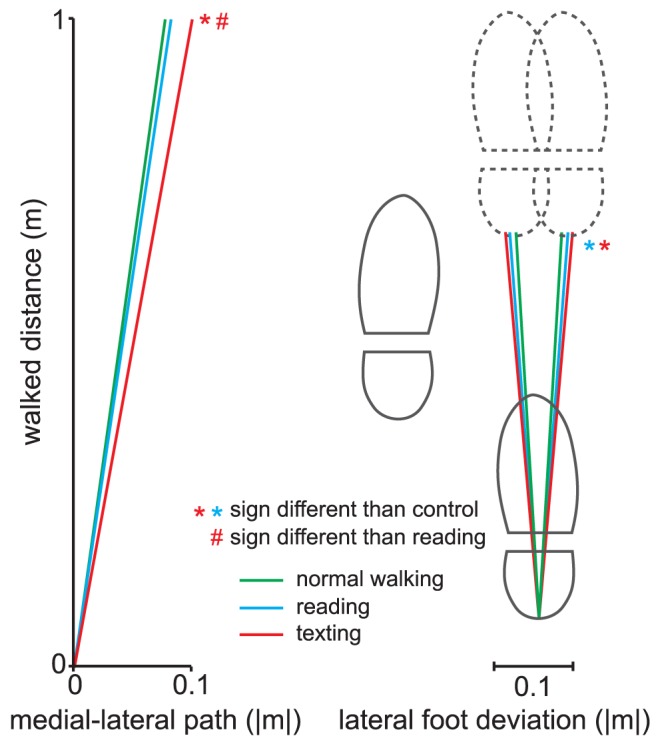
Lateral deviations while walking. The left hand side depicts the absolute medial-lateral deviations from the straight line. The right hand side depicts the absolute change in lateral foot position from one stride to the next of the right foot. The absolute change in lateral foot position per stride was greater during reading and texting than walking, but did not differ between the two phone tasks.

Participants deviated more from a straight line during reading and texting than during the walking task (Table 2). The summed absolute distance in lateral direction per meter walked was greater during texting than reading on a mobile phone or normal walking. The absolute change in lateral foot position per stride was greater during reading and texting than walking, but did not differ between the two phone tasks (Fig. 2).

### Coordination of the head and thorax (segment angles and phase angle)

**Figure 3:**
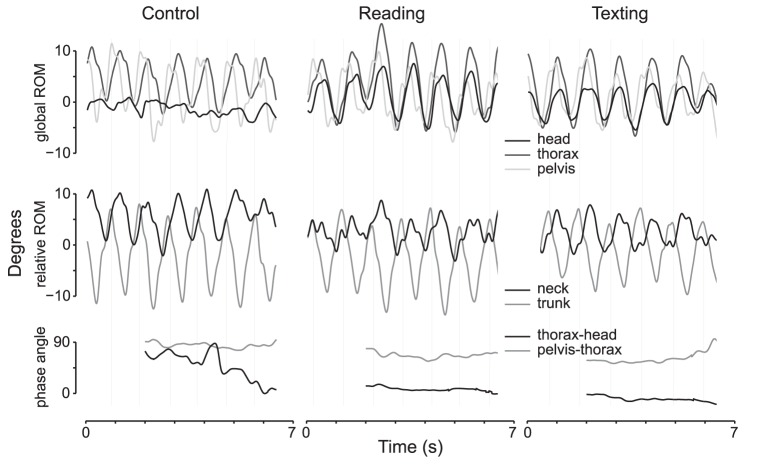
Example of rotation motion of pelvis, thorax and head, and the relative rotation motion between thorax and head (neck) and pelvis and thorax (trunk) and phase angle between thorax-head and pelvis-thorax rotations when a participant walked without a phone (Control), read on a mobile phone (Reading) and texted on a mobile phone (Texting). Note the increase in range of head rotation in relation to the global reference frame during reading and texting with reduction of phase angle and phase variability between thorax and head. The dashed vertical grey lines denote right heel strikes.

Participants looked at their phone for reading and texting with a flexed head position, and the angle of flexion did not differ between these conditions (see Table 3 for data and statistical analyses). In the global frame of reference, head flexion-extension ROM was less during reading and texting than walking without the phone and less during texting than reading (Fig. 3). In contrast, head lateral flexion ROM was greater during reading than walking but texting was not different from reading or walking without a mobile phone. Head rotation was greater during reading and texting than walking, but did not differ between reading and texting. Thorax flexion-extension ROM was less during reading and texting than walking, and lower during texting than reading. Thorax lateral flexion ROM decreased more during texting, than reading and walking. Rotation of the thorax did not differ between conditions. The flexion-extension, lateral flexion and rotation ROM of the neck reduced during reading and texting compared to walking, and was lower during texting than reading (Table 3). This finding concurs with a more “in-phase” thorax-head phase relationship (i.e. smaller phase angle) in all planes during reading and texting than walking. Variability of the relative phase angle between head and thorax lateral flexion and rotation motion was lower in texting and reading than walking (Fig. 3). There was a tendency, although non-significant, for a similar change in flexion-extension motion.

**Table 3:** Segment angular range of motion (°).

	ANOVA_RM_		Post hoc analyses			Mean (±SD)		
Outcome measure	F ratio	P-value	Walk vs. Read	Walk vs. Text	Read vs. Text	Walk	Read	Text
Head flexion position	168.06	0.0000	0.0000	0.0000	0.1881	0.47 (5.63)	29.22 (9.12)	31.80 (10.76)
**ROM in global axis**								
Head flexion-extension	44.3	0.0000	0.0001	0.0000	0.0001	6.65 (2.04)	5.20 (1.46)	4.20 (1.47)
Head lateral flexion	8.35	0.0015	0.0005	0.1609	0.1196	4.51 (1.74)	6.06 (2.57)	5.23 (2.15)
Head rotation	12.47	0.0003	0.0000	0.0048	0.3849	4.75 (1.67)	6.57 (2.75)	5.96 (2.56)
Thorax flexion-extension	19.59	0.0000	0.0018	0.0000	0.0411	3.78 (1.18)	3.38 (1.11)	3.06 (0.74)
Thorax lateral flexion	13.34	0.0000	0.2776	0.0000	0.0045	5.51 (2.11)	4.90 (1.38)	4.20 (1.38)
Thorax rotation	2.96	0.0762				6.33 (1.56)	7.26 (2.41)	7.00 (2.40)
Pelvis flexion-extension	5.05	0.0132	0.7079	0.0083	0.1711	6.68 (4.25)	5.90 (2.76)	5.35 (2.17)
Pelvis lateral flexion	31.29	0.0000	0.0002	0.0000	0.0029	12.14 (4.05)	10.79 (3.97)	9.70 (3.23)
Pelvis rotation	10.62	0.0008	0.0037	0.0002	1.0000	15.73 (8.11)	11.59 (5.20)	10.49 (3.89)
**Relative ROM**								
Neck flexion-extension	45.35	0.0000	0.0000	0.0000	0.0001	7.05 (2.37)	5.10 (1.68)	3.92 (1.64)
Neck lateral flexion	28.55	0.0000	0.0005	0.0000	0.0033	5.63 (1.97)	4.07 (1.54)	3.11 (1.04)
Neck rotation	21.38	0.0000	0.0212	0.0000	0.0016	5.41 (1.50)	4.60 (1.30)	3.68 (1.06)
Trunk flexion-extension	6.7	0.0045	0.2720	0.0018	0.1767	7.64 (4.66)	6.63 (2.85)	5.99 (2.45)
Trunk lateral flexion	23.35	0.0000	0.0098	0.0000	0.0014	15.72 (4.69)	14.08 (3.87)	12.10 (3.68)
Trunk rotation	40.28	0.0000	0.0003	0.0000	0.0001	16.89 (8.99)	13.77 (6.44)	11.01 (4.86)

### Coordination of the pelvis and thorax (segment angles and phase angle)

ROM of anterior and posterior tilt of the pelvis (sagittal plane) and flexion-extension ROM between the pelvis and thorax were similar during reading and walking, but reduced during texting. In the global frame of reference, pelvic lateral flexion (frontal plane) ROM was lower during reading, and further reduced during texting, when compared to walking. Pelvic rotation (transverse plane) ROM was lower during reading and texting than walking. The rotation ROM of the trunk was lower during reading and texting than walking and ROM during texting was lower than reading, consistent with tighter mechanical constraint between these segments when manipulating the phone in the hands. The flexion-extension phase angle between the pelvis and thorax was less during reading than walking, and lateral flexion phase angle was reduced to a greater extent during texting than reading or walking (Table 4). In both planes, phase angle variability was greater during texting than walking. Phase angle and phase angle variability of pelvis and thorax rotations were unaffected by condition.

**Table 4:** Phase angle (°).

	ANOVA_RM_		Post hoc analyses			Mean (±SD)		
Outcome measure	F ratio	P-value	Walk vs. Read	Walk vs. Text	Read vs. Text	Walk	Read	Text
**Phase angle thorax head**								
Flexion-extension	13.09	0.0000	0.0006	0.0001	1.0000	90.11 (37.17)	57.09 (32.86)	51.35 (32.42)
Lateral flexion	18.92	0.0000	0.0000	0.0000	1.0000	51.21 (33.00)	22.28 (19.66)	20.21 (13.55)
Rotation	9.24	0.0010	0.0121	0.0004	0.7707	34.99 (28.02)	18.97 (17.91)	14.06 (12.47)
**SD phase angle thorax head**								
Flexion-extension	3.77	0.0342	0.0548	0.0754	1.0000	76.41 (20.07)	64.74 (17.98)	65.37 (19.96)
Lateral flexion	19.56	0.0000	0.0000	0.0000	1.0000	28.68 (21.75)	12.88 (10.16)	13.04 (11.38)
Rotation	17.58	0.0000	0.0000	0.0000	1.0000	21.53 (9.48)	13.46 (12.72)	11.96 (8.26)
**Phase angle pelvis thorax**								
Flexion-extension	4.44	0.0185	0.0137	0.6256	0.2882	97.69 (47.16)	70.90 (41.51)	86.87 (38.77)
Lateral flexion	11.58	0.0002	0.3228	0.0001	0.0095	133.06 (31.32)	127.03 (35.41)	115.32 (42.23)
Rotation	3.46	0.0691				98.19 (44.70)	96.00 (50.48)	82.05 (48.50)
**SD phase angle pelvis thorax**								
Flexion-extension	4.78	0.0128	0.4817	0.0098	0.3068	51.92 (17.05)	57.09 (18.87)	63.13 (18.65)
Lateral flexion	5.6	0.0089	0.1570	0.0050	0.5605	8.49 (5.44)	9.33 (4.58)	10.47 (4.89)
Rotation	0.34	0.6484				18.32 (11.93)	17.56 (11.43)	16.49 (11.16)

### Additional experiment – The impact of walking speed on gait kinematics with mobile phone use

When the phone was used for reading or texting with walking speed controlled on a treadmill to match that used during the individuals' overground walking and texting conditions, the following variables were found to be altered as a result of mobile phone use and not walking speed (Table 5). When typing a message on a phone, participants had greater absolute lateral foot position deviation (F_condition_ = 8.70, *P* = 0.0098, *P*_posthoc_ = 0.0103) and reduced flexion-extension ROM of the head (F_condition_ = 6.98, *P* = 0.0176, *P*_posthoc_ = 0.0173) when compared with normal walking. Similarly, when reading or typing a message, participants walked with a more flexed head position (F_condition_ = 38.82, *P* = 0.0001, *P*_posthoc_<0.0004), greater rotation ROM of the head in the global reference frame (F_condition_ = 20.64, *P* = 0.0007, *P*_posthoc_<0.0036), less neck rotation ROM (F_condition_ = 6.92, *P* = 0.0180, *P*_posthoc_<0.0432) and the phase angle between thorax and head rotations that was more in-phase (F_condition_ = 10.91, *P* = 0.0052, *P*_posthoc_<0.0124) compared with normal walking. In contrast to the overground experiment, the phase angle between pelvis and thorax lateral flexion increased when manipulating a phone compared to walking without a phone (F_condition_ = 11.16, *P* = 0.0048, *P*_posthoc_<0.0111).

**Table 5:** Additional treadmill experiment data.

	Normal walking speed			Walking speed while texting		
Outcome measure	Walk	Read	Text	Walk	Read	Text
Delta right foot position (m/stride)	0.015 (0.002)	0.018 (0.003)	0.020 (0.003)	0.017 (0.003)	0.017 (0.002)	0.019 (0.003)
Head flexion position (°)	2.67 (1.96)	27.22 (6.72)	31.92 (8.68)	2.38 (2.25)	27.46 (7.62)	32.37 (10.44)
**ROM in global axis (°)**						
Head flexion-extension	4.59 (1.35)	4.22 (0.61)	3.46 (0.40)	4.64 (0.79)	3.98 (0.47)	3.50 (0.58)
Head rotation	3.77 (1.40)	6.94 (2.00)	6.40 (1.38)	4.80 (1.36)	7.03 (2.04)	6.54 (1.18)
**Relative ROM (°)**						
Neck rotation	5.98 (1.99)	4.79 (1.32)	4.45 (1.41)	6.24 (2.42)	4.13 (1.11)	4.26 (0.92)
**Phase angles (°)**						
Thorax head rotation	28.04 (7.95)	15.85 (11.49)	15.15 (2.60)	21.51 (5.75)	10.56 (1.51)	10.41 (5.42)
Arm swing thorax rotation	45.96 (27.02)	17.36 (6.45)	15.37 (7.47)	29.45 (20.57)	10.92 (5.36)	12.02 (5.17)
**Phone movement (path, m/sec)**						
irt Global frame		0.079 (0.012)	0.073 (0.009)		0.068 (0.012)	0.059 (0.005)
irt Head frame		0.039 (0.011)	0.036 (0.015)		0.033 (0.008)	0.032 (0.011)

Data (mean ± standard deviation) are shown for the additional treadmill experiment. The outcome measures that were affected by mobile phone use and not walking speed are shown.

The additional analysis revealed that the phase angle between the forward-backward motion of the elbow and rotation of the thorax was smaller (moved almost ‘in-phase’), when participants manipulated the phone for reading or texting while walking on a treadmill than walking without a phone (F_condition_ = 7.55, *P* = 0.0144, *P*_posthoc_<0.0310). This finding suggests the phone was more ‘connected’ with the thorax and confirms our observations from the overground walking experiment. Movement of the phone (path, m/s) with respect to the head reference frame was less than that observed in the global reference frame (F_reference frame_ = 20.76, *P* = 0.0104), but did not differ between texting and reading (F_reading_texting_ = 2.71, *P* = 0.1752). These results confirm that head motion is closely linked to thorax motion and this is likely to reduce movement of the phone in the visual field.

## Discussion

This study is the first to compare the impact of typing text on a mobile phone on gait performance and kinematics against that associated with reading text on a phone and walking without constraint, and without any additional restriction of field of view. Evaluation of gait performance revealed that individuals walk slower, demonstrate greater absolute medial-lateral step deviation, increase rotation ROM of the head with respect to the global reference frame, walk with a flexed head position, reduce neck ROM, and move the thorax and head more in-phase with reduced phase variability, during texting and reading than unconstrained walking. Differences between typing and reading text were less pronounced, but typing text was associated with slower walking speed, greater deviation from a straight line, more ‘in-phase’ lateral flexion motion between the thorax and pelvis and generally reduced ROM of the neck compared to reading text on a mobile phone. Furthermore, while reading, phase angle between pelvis and thorax flexion-extension was reduced. These findings are similar to those observed in previous studies. For instance, Lamberg and Muratori [8] reported reduced walking speed, increased lateral deviation and an increase in the distance travelled during texting. Similarly, Demura and Uchiyama [4] showed reduced walking speed and stride width when using the email function on a mobile phone. Our data indicate that typing text, and to a lesser extent reading text, on a mobile phone impairs gait quality. Taken together with the observation that 35% of our participants reported previous accidents while typing text, these data could be interpreted to suggest texting may pose an additional risk to safety when pedestrians are required to navigate obstacles or cross a road.

As participants walked slower while reading and reduced speed further while texting, some changes in gait kinematics may be explained by reduced speed. The additional experiment performed on a treadmill was conducted to evaluate this confounding effect. Participants walked at their normal (control) and texting speed, derived from the overground walking experiment. The following variables were less likely to be affected by reduced speed, and more likely to be related to the effect of dual tasking with a phone: 1) phone movement closely related to head movement, which likely makes it easier to read or type a message on a phone and 2) motion of the arms was closely related to thorax rotation, which is likely to reduce the number of degrees of freedom controlled by central nervous system. The resultant coupling of motion of the arms (and phone), thorax and head would maintain the phone in a steady position in the visual field. Although the reduced phase angle and almost in-phase coordination between head and thorax rotation would facilitate steadiness of the phone for reading, this has negative consequences, as head stability in the global reference frame is compromised. This strategy to optimise the phone task, may compromise the accuracy of head control and impact on balance performance. This hypothesis is supported by increased medial lateral head motion of ∼1.5 degrees during texting and reading in the current study which, although small, exceeds the threshold for detection of sway with proprioceptive, visual and vestibular systems in humans [12], thus adding noise to balance information. Increased medial-lateral head motion is associated with a greater risk of falling in healthy older adults [13] and individuals with Parkinson's disease [14]. Further, young healthy adults are known to adopt a preferred walking speed, step length and cadence in order to optimise stability of the head [15]. Reduced walking speed during reading and texting could be an attempt to minimize movements of the head in space. The increased demand associated with manipulating a mobile phone may cause young healthy adults to prioritise movement of the head relative to the trunk at the expense of gait stability. This may underpin increased medial-lateral deviation of heel strikes, greater deviation from a straight path (while texting), and increased phase angle variability between pelvis and thorax (flexion-extension and lateral flexion directions) in the current study. Higher variability of relative phase angles may increase the potential for internal (i.e. self-generated) perturbations to balance, and negatively affect gait stability.

A key finding was reduced neck ROM (head relative to thorax) in all planes during reading, and to a greater extent with typing text. The head moved more ‘in-phase’ with the thorax, and coordination between segments was less variable (lower phase angle variability) in lateral flexion and rotation directions. These findings imply the head is controlled in a manner that constrains its relationship with the thorax, most likely to optimize the relationship between the eyes, trunk/arm and phone. This is supported by our observation that the arms were ‘locked’ to the thorax, such that the phone moved together with the thorax, in the overground experiment and confirmed in the treadmill experiment where forward-backward arm swing shifted to an almost ‘in-phase’ relationship with thorax rotation. Motion of the arm was more ‘out-of-phase’ when walking on a treadmill without the phone. Phone movement with respect to the head frame of reference was lower than in the global frame of reference. Reduced arm swing can negatively impact on walking balance. For instance, arm swing reduces angular momentum about the vertical axis [16], reduces the metabolic cost of walking [17], and assists with recovery after disturbance to walking balance [18], [19]. Reduced walking speed with phone use could partially be explained by reduced arm swing, as arm swing can compensate for increased angular momentum that occurs with increased walking speed [16]. Further investigation is required to explore changes in angular momentum with phone use during walking and its potential additional effect on recovery after perturbation to walking balance.

Changes in gait associated with mobile phone use may undermine functional walking and impact on safety in common pedestrian environments. Individuals with constrained movement patterns [20], slower walking speeds [20], and those who perform a cognitive task while walking (often referred to as dual-tasking) are at greater risk of collisions or falls [21]. Dual-tasking competes for cognitive resources and can lead to prioritisation of one task [22]–[25]. Although contemporary theories suggest a ‘posture first’ strategy in healthy individuals that prioritises gait stability over a cognitive task [26], recent work has challenged this theory leading to the proposal that cognitive tasks may be prioritised based on postural reserve, hazard estimation, expertise and task complexity. In this model healthy individuals can elect to prioritise the cognitive task over gait stability when there is sufficient safety margin [25]. Our data support this proposal as young healthy individuals prioritised typing or reading text (a cognitive task) over optimisation of walking, with a consequent compromise to balance and its stability. This compromise was tolerated in the predictable research environment, but could be problematic in the face of unexpected challenges to gait. In the present study we did not assess participants' ability to dual task or stratify our sample based on this skill. However, ability to dual-task is known to vary between individuals [27]. It is possible that those with good ability to dual-task may exhibit better gait performance during phone manipulation than those with poor ability to perform dual-tasks. Future studies should seek to explore the relationship between ability to dual task, history of accidents and gait performance during mobile phone use.

The gait kinematic most likely to impact on safety was the deviation from a straight walking path during typing and reading text on a mobile phone. In a pedestrian environment inability to maintain a straight path would be likely to increase potential for collisions, trips and traffic accidents. There are two plausible mechanisms for the inability to maintain a straight walking path during texting and reading. First, reduced awareness of the visual field would limit use of external cues to guide path, and second the greater head motion relative to the global reference frame (but greater constraint to the trunk) may reduce the utility of vestibular information. Vestibular input is essential for accurate navigation during walking [28] and alteration of head posture impacts an individual's ability to accurately interpret vestibular information for balance [28]. The flexed head posture and greater head motion relative to the external world (global reference frame) adopted by participants when typing and reading text would introduce ‘noise’ into the vestibular information and could interfere with the individual's ability to accurately navigate a straight walking path.

## Conclusion

This study is the first to compare the impact of typing and reading text on a mobile phone on gait performance. We demonstrate slower walking speed, greater deviation from a straight path and increase absolute lateral step deviation in conjunction with increased rotation ROM of the head in global space, reduced relative motion and greater ‘in-phase’ motion of the head during typing, and to a lesser extent, reading text on a mobile phone than normal walking. These altered gait parameters may have an impact on the safety of pedestrians who type or read text on a mobile phone while walking.
