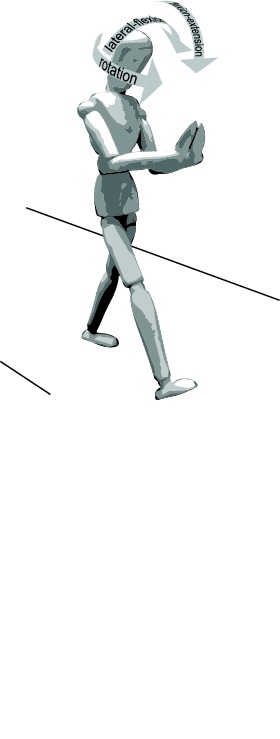# Correction: Texting and Walking: Strategies for Postural Control and Implications for Safety

**DOI:** 10.1371/journal.pone.0091489

**Published:** 2014-02-28

**Authors:** The PLOS ONE Staff

Figure 1 is distorted. This error occurred while the article was being prepared for publication. Please see the correct Figure 1 here:

**Figure:**